# Correlation Study between the Organic Compounds and Ripening Stages of Oil Palm Fruitlets Based on the Raman Spectra

**DOI:** 10.3390/s22187091

**Published:** 2022-09-19

**Authors:** Muhammad Haziq Imran Md Azmi, Fazida Hanim Hashim, Aqilah Baseri Huddin, Mohd Shaiful Sajab

**Affiliations:** 1 Department of Electrical, Electronic and Systems Engineering, Faculty of Engineering and Built Environment, Universiti Kebangsaan Malaysia, Bangi 43600, Selangor, Malaysia; 2 Research Centre for Sustainable Process Technology (CESPRO), Faculty of Engineering and Built Environment, Universiti Kebangsaan Malaysia, Bangi 43600, Selangor, Malaysia; 3 Department of Chemical and Process Engineering, Faculty of Engineering and Built Environment, Universiti Kebangsaan Malaysia, Bangi 43600, Selangor, Malaysia

**Keywords:** Fresh Fruit Bunches (FFB), Raman spectroscopy, machine learning, ripening, oil palm fruitlets

## Abstract

The degree of maturity of oil palm fresh fruit bunches (FFB) at the time of harvest heavily affects oil production, which is expressed in the oil extraction rate (OER). Oil palm harvests must be harvested at their optimum maturity to maximize oil yield if a rapid, non-intrusive, and accurate method is available to determine their level of maturity. This study demonstrates the potential of implementing Raman spectroscopy for determining the maturity of oil palm fruitlets. A ripeness classification algorithm has been developed utilizing machine learning by classifying the components of organic compounds such as β-carotene, amino acid, etc. as parameters to distinguish the ripeness of fruits. In this study, 47 oil palm fruitlets spectra from three different ripeness levels—under ripe, ripe, and over ripe—were examined. To classify the oil palm fruitlets into three maturity categories, the extracted features were put to the test using 31 machine learning models. It was discovered that the Medium, Weighted KNN, and Trilayered Neural Network classifier has a maximum overall accuracy of 90.9% by using four significant features extracted from the peaks as the predictors. To conclude, the Raman spectroscopy method may offer a precise and efficient means to evaluate the maturity level of oil palm fruitlets.

## 1. Introduction

One of the major economic drivers of the agricultural sector in developing nations such as Malaysia and Indonesia is the palm oil business [1]. The enormous market demand for palm oil products, including for consumption, the cosmetics sector, and many other uses, makes the quality of Fresh Fruit Bunch (FFB) palm oil an important factor. Finding the correct fruit to harvest is one of the biggest issues facing the fruit industry. Oil palm FFB must be harvested at the proper stage to retain and protect the product’s quality. The level of post-harvesting determines the palm oil industry’s profitability. Therefore, optimal quality is ensured and oil output is maximized by harvesting FFB oil palm at the proper stage of maturity [2].

The traditional approach to agricultural product quality assessment is typically time-consuming and expensive. Traditional methods have been used for a very long time, but they are extremely tedious, expensive, and time-consuming [3]. The experience, abilities, and emotional state of the harvester have a significant impact on the ability to harvest FFB based on visual observation. Visually assessing the harvest is made more challenging by the placement of the fruit, the height of the trees, and the angle of the sun [4]. Currently, color and loose fruitlets inspection are utilized to determine the matured stage. When the fruitlets’ mesocarp’s outermost layer develops a yellowish-orange color and around 10 fruitlets that have fallen out of their sockets, the FFB is prepared for harvest [5]. The manual grading of oil palm FFBs is a labor-intensive, time-consuming operation that is vulnerable to human errors and biased evaluation, which has a significant negative impact on the growers’ profitability. As a result, a quick, trustworthy, and precise grading method is required to identify oil palm FFB ripeness [6].

Various computer vision studies have recently been investigated for the classification of ripeness in fruits such as watermelon, bananas, and tomatoes. Most of these studies extract useful information about the spectral characteristics of the fruit surface by using color as a measure to evaluate the stage of ripeness [2]. This method has a drawback in that changes in sunlight intensity have a substantial impact on item color. Therefore, the tool for forecasting fruit quality, ripeness, and harvest should not be influenced by surrounding light conditions [4].

The ripeness of tomatoes and citrus fruits have recently been proved by researchers using Raman spectroscopy to measure the molecular vibrations of organic compounds contained in the fruits’ skins [7,8]. Both a portable Raman spectrometer and a confocal Raman spectrometer were used to demonstrate these investigations. High intensity light is employed by a Raman-based device to measure the inelastic scattering from the surface of a target molecule, which is then used as the compound’s molecular fingerprint [9]. All studies have demonstrated that the Raman shifts, which are thought to represent important organic compounds such as carotenoids, chlorophyll, etc. may be linked to molecular vibrations. However, previous research has revealed that each of these raw Raman spectra is composed of convoluted signals produced by various organic molecules [9]. This spectrum’s deconvolution and additional signal synthesis may enable a more precise identification of the organic compounds contained in the fruit.

This study examines the organic properties of the exocarp of oil palm fruitlets using confocal Raman spectroscopy. The raw Raman spectroscopy of the fruitlets will then be pre-processed further and deconvoluted to identify organic compounds such as β-carotene, cuticular wax, chlorophyll-a, etc. To automatically determine the level of maturity of the oil palm fruitlets, these components are retrieved as predictors for our classification model based on multiple models of machine learning.

## 2. Materials and Methods

### 2.1. Oil Palm Fruit Samples Preparation

The National University of Malaysia (UKM) owns the oil palm farm, which is run by its commercial branch, JANA@UKM. This is where the oil palm fruit samples were collected. The samples are from the Elaeis guineensis DxP species, a hybrid between the thick-shelled Elaeis guineensis fo. dura and the shell-less Elaeis guineensis fo. pisifera species. The ripeness levels for under ripe, ripe, and over ripe fruitlets were determined using the Malaysian Palm Oil Board (MPOB) grading standard as a guideline [5]. Since the under ripe fruit is hard and intact, it was chipped out of the bunch. The over ripe fruits were obtained from detached fruitlets, while the ripe fruits were taken from a harvested bunch. An in-house grader from JANA@UKM examined and validated each fruitlet. A total of 47 samples, including 13 under ripe, 19 ripe, and 15 over ripe samples, were gathered. Fresh fruitlets were collected, checked, and labeled to be transported to the lab for sample preparation. For Raman scanning, the oil palm fruit samples were made into a thin layer. To do this, a thin layer of the oil palm fruitlet’s exocarp (skin) was cut off using a scalpel, placed to a microscope slide, and examined with a Raman microscope.

### 2.2. Raman Instrumentation

A tabletop confocal Raman spectrometer was used to gather the Raman spectra (Thermo Scientific, DXR2xi Raman Imaging Microscope, Waltham, MA, USA). A 532 nm laser, 900 lines/mm grating, 50 m slit aperture, and green filter, were used during the spectroscopy analysis. The samples were irradiated three times for a duration of 3 milliseconds, each using a 2.0 mW laser. The top, middle, and bottom of the fruitlet were the three separate locations from which the spectra for each sample were taken.

### 2.3. Raman Spectra Pre-Processing

To reduce noise in the raw spectra collected from the Raman spectrometer, the raw Raman spectra were then treated using signal processing techniques. Rubber band algorithm was used to correct the baseline for the spectra first. Any zero offsets in the spectra were eliminated in this step. Next, a smoothing process was applied to the baseline-corrected spectra. The Savitzky–Golay filter in second order with a 21-point filter size was used to achieve this. This method makes sure that any high-frequency noise in the spectra is removed. Next, the entire spectra were segmented into two areas: 920 to 1040 ${\mathrm{cm}}^{-1}$ and 1370 to 1460 ${\mathrm{cm}}^{-1}$, which later will be referred as to Area 1 and Area 2, respectively. Both segmented spectra areas were then subjected to a deconvolution procedure utilizing the Gaussian curve fitting methods [9,10,11]. The deconvolution process was carried out to extract organic compound characteristics from the Raman spectrum via OriginPro 2021. Then, multiple features were extracted from each deconvoluted peak such as peak’s intensity (height), peak’s position, peak’s the full width at half maximum (FWHM), and peak’s area. These features were then tested in a statistical analysis to investigate their significance in determining the ripeness of the fruitlets.

### 2.4. Statistical Analysis

Statistical analysis was conducted using IBM SPSS Statistics software to investigate the significance of each feature that has been extracted from the deconvoluted peak towards evaluating the ripeness of FFB. The one-way ANOVA, homogeneity test, and post hoc test are the statistical methods used in this study. To find the variance difference between the three ripeness groups, a homogeneity test was utilized. If the variances were equal, the Gabriel test was performed. However, if the variances were unequal, a Welch test was conducted to validate the findings. To examine the means of the chosen features, one-way ANOVA was then performed. This analysis aids in eliminating features that have little contribution in determining the ripeness of an oil palm fruitlets. A post hoc test and a multi comparison analysis were then performed to determine the features that significantly contribute to the differentiation of ripeness. The validity test of the features found uneven variance, therefore a second Games–Howell test was performed. The significance of a feature depends on the *p*-value extracted from the Gabriel/one-way ANOVA test, where a *p*-value greater than 0.05 shows that the feature is not significant. After all these tests were performed, the significance of each feature was observed and features that were not significant were eliminated, while the significant features were used in classification model development.

### 2.5. Classification Analysis

The significant features from the result of the statistical analysis were used as predictors in building the classification model based on machine learning. A total of 31 machine learning models including K-Nearest Neighbor (KNN), Support Vector Machine (SVM), Neural Network, etc., were trained using the predictors extracted, using MATLAB by Mathworks. The classification analysis used implements the 5-fold cross validation to protect against overfitting. This means that the given dataset was split into five equal portions and iterated five times to find its performance. At each iteration, 1 portion was used as the test data, while the other four portions were used as the train data. A k-fold cross validation was expected to give a more accurate estimate of the model’s mean performance. The performance score or accuracy for each classifier was calculated and recorded. The process was repeated by including and excluding some features or predictors to achieve the maximum accuracy score possible.

## 3. Results

### 3.1. Raman Spectra Pre-Processing

**Figure 1:**
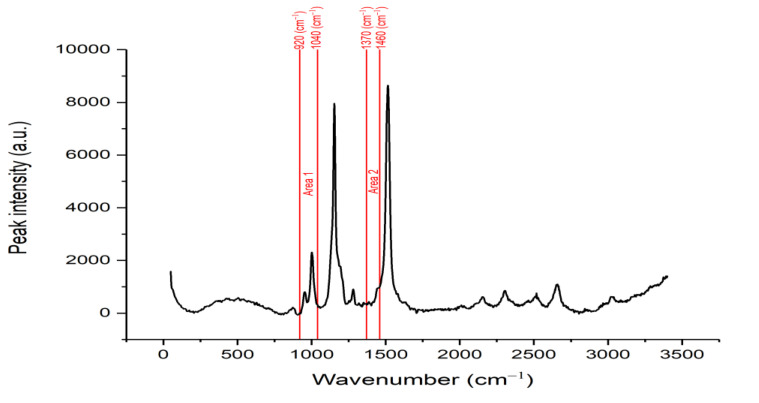
Raw Raman spectra of an oil palm fruit.

The whole raw Raman spectrum of an oil palm fruit is shown in Figure 1. The raw Raman spectrum contained a lot of noise and zero offset, which had the potential to cause problems in the deconvolution process as well as analysis process later, as we needed to implement the curve fitting method on the spectrum. Elimination of this noise and zero offset was required to produce a good peak deconvolution result. By applying baseline correction with the addition of the Savitzky–Golay filter, the spectrum had less noise.

**Figure 2:**
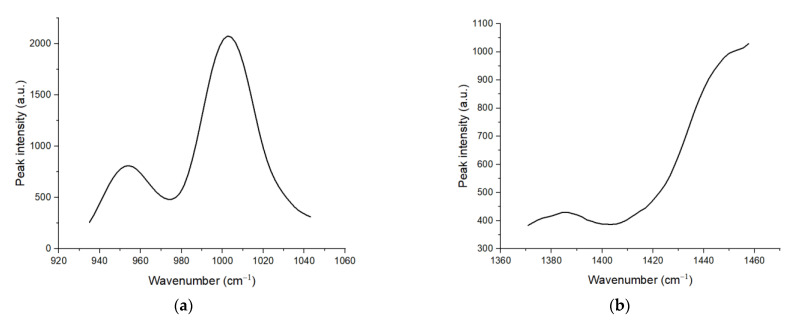
(**a**) Segmented Area 1 (920 to 1040 ${\mathrm{cm}}^{-1}$); (**b**) Segmented Area 2 (1370 to 1460 ${\mathrm{cm}}^{-1}$).

**Figure 3:**
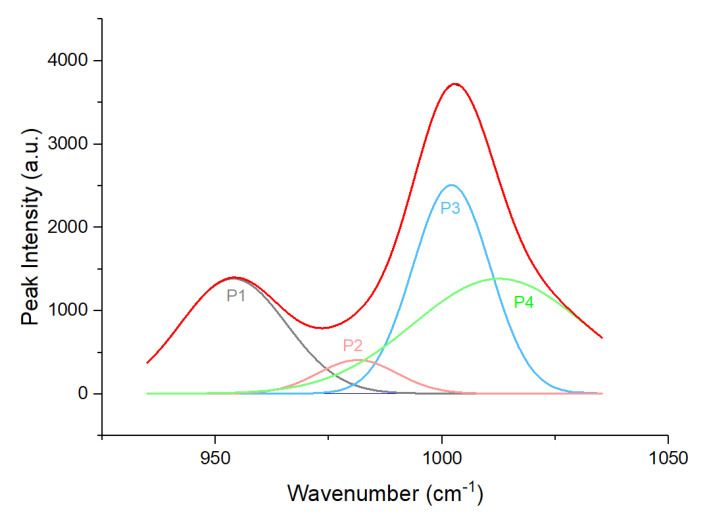
Deconvolution results of Area 1 (920 to 1040 ${\mathrm{cm}}^{-1}$) with P1 (1st peak), P2 (2nd peak), P3 (3rd peak) and P4 (4th peak).

**Figure 4:**
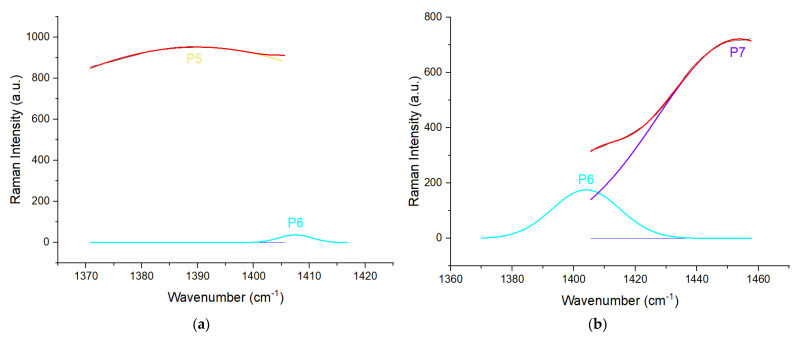
(**a**) Deconvolution results of left side of Area 2 with P5 (5th peak) and P6 (6th peak); (**b**) Deconvolution results of right side of Area 2 with P6 (6th peak) and P7 (7th peak) (1370 to 1460 ${\mathrm{cm}}^{-1}$).

As mentioned before, we focused on two areas: 920 to 1040 cm^−1^ (Area 1) and 1370 to 1460 cm^−1^ (Area 2). Therefore, we needed to segment these two areas from the pre-processed spectra. The segmented Area 1 and Area 2 are shown in Figure 2a,b, respectively. Based on these two segmented spectra, the deconvolution process was carried out on these peaks to obtain hidden peaks, in which each hidden peak represents an organic component. However, on Area 2, the spectrum was divided into two sides: left and right, and was deconvoluted individually, where peak fitting is performed on the left and right peaks separately to find hidden peaks. The reason for this was to reduce errors while performing peak fitting on the whole area with multiple peaks. In Area 1, a total of four peaks with distinct peak position and intensities were found, while a total of four peaks were found in Area 2, as shown in Figure 3 and Figure 4a,b, respectively. Four peaks were found in Area 2; however, after further investigation, the 2nd peak on the left side was the same as the 1st peak on the right side. Hence, both peaks could be counted as a single peak.

In Area 1, the hidden peaks were located at 954, 980, 1002, and 1017 cm^−1^ on average. Using the findings from [12,13,14,15,16,17,18], we determined that peaks averaged at 954, 980, 1002, and 1017 cm^−1^ were β-carotene, chlorophyll-a, β-carotene, and amino acid, respectively. In Area 2, the hidden peaks were located at 1387, 1409, and 1454 cm^−1^ on average. Based on findings from [12,14,15,16,17,18], peaks averaged at 1387, 1409, and 1454 cm^−1^ were determined to be β-carotene, cuticular wax, and β-carotene, respectively. A summary of the molecular assignment for the peaks is presented in Table 1 and Table 2.

### 3.2. Features Extraction and Analysis

In the feature analysis, we extracted four crucial Raman spectral feature components that could be used to accurately determine the ripeness of an oil palm fruit The peaks’ positions, the peaks’ intensities, FWHM of the peaks and the peaks’ areas are the features that can be extracted from the deconvoluted peaks.

The peak position is the feature that is controlled first by the isolated molecule’s natural vibrational frequency [25], which shows the phase or stoichiometric composition of the substance [26].

**Figure 5:**
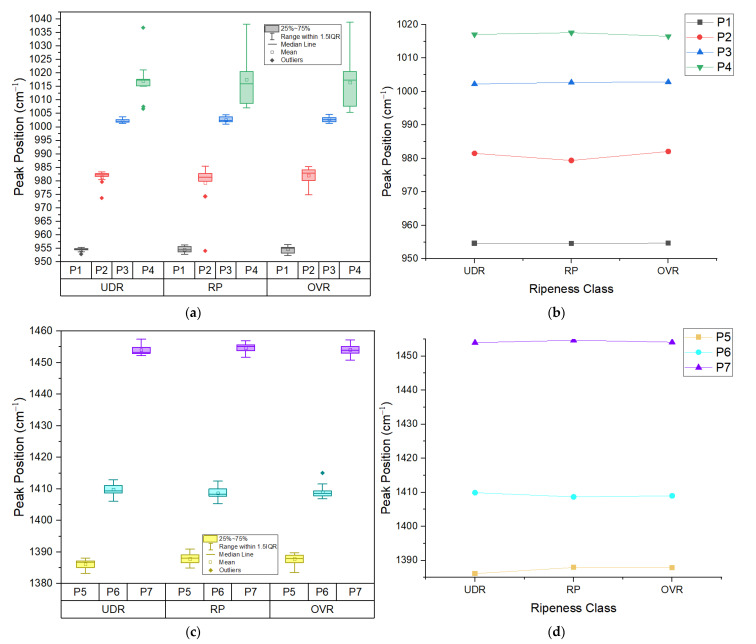
(**a**) Grouped box plot and (**b**) mean plot of peak’s position of 4 peaks in Area 1; (**c**) Grouped box plot and (**d**) mean plot of peak’s position of 3 peaks in Area 2. UDR: Under ripe, RP: Ripe, OVR: Over ripe; P1: β-carotene, P2: chlorophyll-a, P3: β-carotene, P4: amino acid, P5: β-carotene, P6: cuticular wax, P7: lycopene/β-carotene.

Figure 5a,c each show a grouped box plot of extracted peak position for three levels of ripeness for Area 1 and Area 2, respectively. Based on the box plot, there are small changes in box plot size from UDR (under ripe) to OVR (over ripe) level in peak position for all peaks except for Peak 4 (amino acid), where the size of the box plot increases immensely as the ripeness level increases. The smaller box plot size indicates that there is a small variation within the sample group that is being measured. Moreover, two outliers are found in Peak 2 in UDR and RP state, three outliers in Peak 3 in UDR state, and one outlier was found in Peak 6 in OVR level. The mean values for each peak were fed through the Gabriel and Games–Howell tests to test for significance between ripeness groups. Figure 5b,d, respectively, shows the significant difference between the groups through the mean plot of the peak’s positions. According to the statistical tests (and as presented in Figure 5b,d), all peaks do not show any significant difference between UDR and RP, RP and OVR, and UDR and OVR, except for Peak 5 (β-carotene), which shows significant difference between UDR and RP, and UDR and OVR. We can say that the peak positions in general are not a good indicator for ripeness levels.

**Figure 6:**
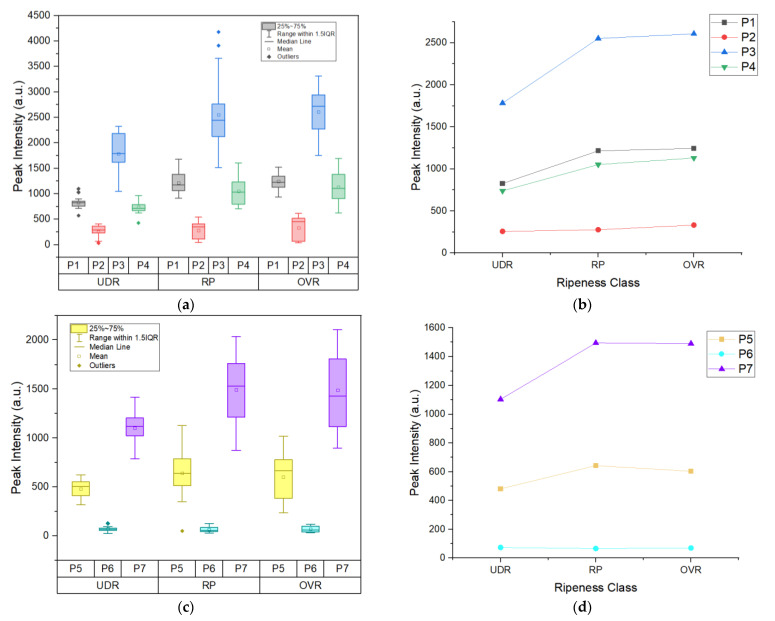
(**a**) Grouped box plot and (**b**) mean plot of peak’s intensity of 4 peaks in Area 1; (**c**) Grouped box plot and (**d**) mean plot of peak’s intensity of 3 peaks in Area 2. UDR: Under ripe, RP: Ripe, OVR: Over ripe; P1: β-carotene, P2: chlorophyll-a, P3: β-carotene, P4: amino acid, P5: β-carotene, P6: cuticular wax, P7: lycopene/β-carotene.

The next feature is the peak intensity with the unit of arbitrary (a.u.). A peak’s intensity is determined by the concentration of molecules present and the strength of absorption (absorptivity) [25]. Figure 6a,c show the grouped box plot of extracted peaks intensity for three ripeness states for Area 1 and Area 2, respectively. Figure 6b,d, respectively, shows the difference between ripeness states through the mean plot of the peak’s intensity. The mean values of each peak were subjected to statistical testing to identify peaks that show a significant difference between the ripeness states. From both Gabriel and Games–Howell statistical tests, several peak intensities were found to show a significant difference between UDR to RP and UDR to OVR. These peaks are Peak 1 (β-carotene), Peak 3 (β-carotene), Peak 4 (amino acid) and Peak 7 (lycopene/β-carotene). For the Games–Howell test alone, Peak 5 (β-carotene) shows a statistically significant difference between UDR to RP. From here, we can conclude that β-carotene and amino acid indicators from the Raman peak intensities could be useful predictors of the ripeness state.

**Figure 7:**
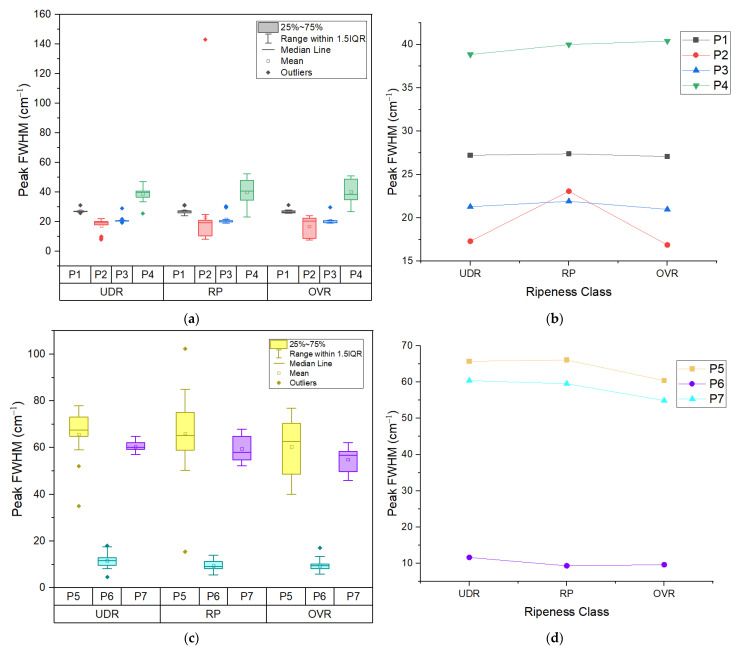
(**a**) Grouped box plot and (**b**) mean plot of peak’s FWHM of 4 peaks in Area 1; (**c**) Grouped box plot and (**d**) mean plot of peak’s FWHM of 3 peaks in Area 2. UDR: Under ripe, RP: Ripe, OVR: Over ripe; P1: β-carotene, P2: chlorophyll-a, P3: β-carotene, P4: amino acid, P5: β-carotene, P6: cuticular wax, P7: lycopene/β-carotene.

The third feature is the peak’s FWHM. FWHM is typically used to report line widths. Despite having the highest information content, the line width is the spectroscopic characteristic that receives the least attention. The line width is impacted by all dynamics such as motion and energy loss [25]. As shown in the box plots in Figure 7a,c and in the mean plots in Figure 7b,d, Peak 1 and 3, both representing β-carotene, have one outlier for every ripeness level and show no variation in values as the ripeness level increases due to their small box plot size. From the statistical tests on the mean values, almost all peaks except Peak 7 do not show any significant difference between the ripeness states. Peak 7 (lycopene/β-carotene) shows a statistically significant difference between RP to OVR and UDR to OVR. So far, only this peak’s FWHM could confidently differentiate between the RP and OVR groups.

**Figure 8:**
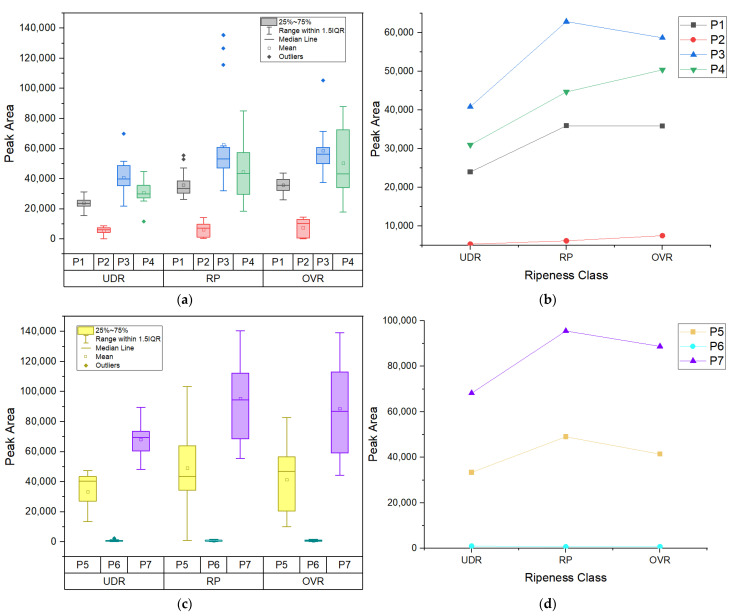
(**a**) Grouped box plot and (**b**) mean plot of peak’s area of 4 peaks in Area 1; (**c**) Grouped box plot and (**d**) mean plot of peak’s area of 3 peaks in Area 2. UDR: Under ripe, RP: Ripe, OVR: Over ripe; P1: β-carotene, P2: chlorophyll-a, P3: β-carotene, P4: amino acid, P5: β-carotene, P6: cuticular wax, P7: lycopene/β-carotene.

The last feature that to be extracted was the peak’s area under the curve. Since the final peak profile is the sum of all the separate components, the peak’s area provides a better measure of concentration. Peak height has traditionally been utilized because it is simpler to measure. However, peak fitting enables the usage of area, which can enhance the calibrations’ linearity [25]. Since a peak’s area is the alternative way of calculating concentration, it was expected for the box plot and trend plot to have the same trend as the peak’s height/intensity, as shown in Figure 8a–d. However, statistical tests on the mean values proved otherwise. Unlike the peak intensity, the peak areas had fewer contributing peaks that showed statistically significant differences between ripeness states. For the Gabriel test, only Peak 1 (β-carotene) showed a statistically significant difference between UDR and RP, and UDR and OVR states. Peak 3 (β-carotene) and Peak 7 (lycopene/β-carotene) showed a significant difference between UDR to RP only, while Peak 4 (amino acid) showed a significant difference between UDR and OVR. For the Games–Howell test, Peak 1 (β-carotene), Peak 3 (β-carotene) and Peak 4 (amino acid) showed a statistically significant difference between UDR and RP, and UDR and OVR groups. Again, the β-carotene and amino acid traces relied predominantly on the Raman peaks for discriminating between ripeness levels.

**Figure 9:**
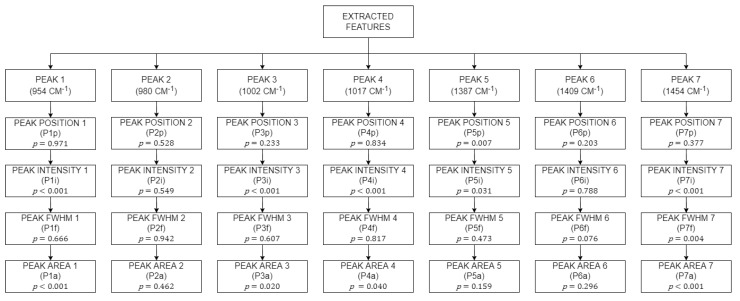
All extracted features from the 7 peaks from both areas including their *p*-values.

All 4 features were extracted from the 7 hidden peaks deconvoluted from both Area 1 and Area 2, which sums up to a total of 28 features that can be extracted. These 28 features were subjected to a thorough statistical analysis to determine their significance in helping to assess fruit’s ripeness level through their *p*-values. The *p*-value gave a value from 0 to 1, with 1 being the difference in outcome purely by chance alone. If the *p*-value was less than 0.05, the feature was deemed statistically significant. Figure 9 shows all the extracted features, where *P1p* represents the peak position for Peak 1, *P1i* represents the peak intensity for Peak 1, *P1f* represents the peak FWHM for Peak 1 and *P1a* represents the peak area under the graph for Peak 1, etc. The *p*-values for all extracted features are included for reference.

### 3.3. Statistical Analysis

A total of 28 features were extracted from the 7 hidden peaks from both Area 1 and Area 2. These 28 features were subjected to homogeneity, Gabriel, Welch, One-way ANOVA, and the Games–Howell test to determine the significance of the feature. Each feature together with its *p*-value are shown in Figure 9.

*P1i*, *P3i*, *P1a*, *P3a*, and *P5p* passed the homogeneity and the one-way ANOVA test. These features were assumed to have equal variance and were significant in discriminating between the ripeness of oil palm fruitlets. The rest of the features that failed the homogeneity test were assumed to have unequal variance. Instead of ANOVA test, features with unequal variance were subjected to the Welch test.

**Figure 10:**
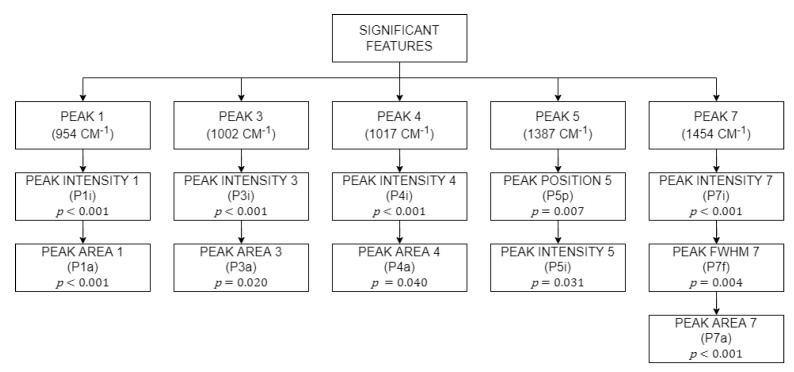
All significant features after statistical analysis.

Subsequently, *P4i*, *P4a*, *P5p*, *P5i*, *P7i*, *P7f*, and *P7a* were found to have failed the homogeneity test. However, since they passed the Welch test, these features were deemed as significant, even though their variance was not equal. A total of 11 features were determined to be significant from a total 28 features extracted earlier. Features that were not significant were eliminated and features that were significant were used as predictors in the machine learning modules later on. All significant features are shown in Figure 10.

### 3.4. Classification Analysis

The 28 features extracted from the deconvoluted peaks earlier have now been reduced to 11 features that were significant after statistical analysis was performed. As mentioned earlier, the classification analysis used implemented the 5-fold cross validation to protect against overfitting. Tabulated in Table 3 are the results for the classification analysis. The first classification analysis was conducted using all 11 features as predictors, showing a maximum accuracy of 81.8% for the Weighted KNN and Discriminant Ensemble classifier. The second classification analysis, however, was performed by including only 4 out of 11 features as predictors to all classifiers. The 4 features are the intensity of Peak 1 and 4 (*P1i* and *P4i*), the position of peak 5 (*P5p*), and FWHM of peak 7 (*P7f*). With only 4 features, the maximum accuracy increased to 90.9% for the Medium KNN, Weighted KNN, and Trilayered Neural Network classifier.

## 4. Discussion

Based on the peak intensity trend discussed earlier, Peak 1 (β-carotene), 3 (β-carotene), 5 (β-carotene), and 7 (lycopene/β-carotene) showed an inclining trend from UDR to RP level. This is due to the fact that Peak 1, 3, 5 and 7 are the molecular assignments for β-carotene, which is predominant in ripe and over ripe levels that contribute to the red-orange pigment in plants which cannot be observed in the under ripe fruitlets, where green pigments dominate [7]. Peak 3 and Peak 7 were also observed to have the highest peak intensity among the other peaks. This finding agrees with previous research [27,28], where β-carotene was claimed to be the highest carotene composition in oil palm fruitlets. The fruit moved closer to maturity as evidenced by the rise in β-carotene peak intensity. Because β-carotene is an orange pigment, the orange pigment’s intensity rises as the fruit matures [9]. It is also easy to distinguish between UDR and OVR using the same peaks. However, differentiating RP and OVR is a bit of a challenge as only Peak 7′s FWHM could differentiate between the RP and OVR group, as statistically proven. However, in reality, farmers are more interested in distinguishing RP from UDR fruits, rather than from RP to OVR fruits, due to harvesting activities.

Subsequently, Peak 6 shows a slight declining trend from UDR to OVR state. This is because Peak 6 represents cuticular wax and based on [7], cuticular wax will show higher intensity in under ripe level compared to other state due to its contribution of skin firmness, consistency and softness, which only can be observed clearly in under ripe plants, fruits and vegetables. The amount of cuticular wax deteriorates as the ripeness level increases. However, according to the statistical tests, Peak 6 does not show enough significant difference between the ripening stages. Peak 4, which represents amino acids, exhibits an inclining trend which agrees with the findings in [29]. Peak 2, which corresponds to chlorophyll-a, shows an inconsistent trend as ripeness increases and failed all the significance tests.

A summary of oil palm ripeness classifiers with their classification techniques, classification algorithm, and accuracy results from previous literature, together with their references, are tabulated in Table 4. Based on Table 4, classifiers that exceed 90% use image processing and hyperspectral techniques. As previously mentioned, the image processing technique is heavily dependent on surrounding lighting and if the lighting is not consistent, such as in oil palm plantation, it may affect the performance of that method. Furthermore, different lighting environments such as sunlight exposure and illumination may require frequent calibration of image capturing devices. For the hyperspectral technique, however, hyperspectral cameras need a lot of storage to save and retrieve the data, hence they are computationally expensive.

## 5. Conclusions

In this study, Raman spectroscopy was used to determine the ripeness of an oil palm fruit, indicating that Raman spectroscopy is an in situ, rapid, and non-invasive method to evaluate the optimum ripeness of FFB. Without cutting or removing the fruits from the plant, Raman spectroscopy is an appropriate technique to observe changes in the composition of oil palm fruit, contrasted by the appearance or disappearance, in addition to the increase or decrease in the intensity of various compounds present in oil palm fruit such as β-carotene, cuticular wax and amino acid, etc. A total of 28 features were extracted and identified through a deconvolution process, with 11 features found to be significant through the statistical analysis process. From these 11 features, 4 were chosen to achieve a classification accuracy of 90.9% using Medium and Weighted KNN and the Trilayered Neural Network.

## Figures and Tables

**Table 1:** Molecular assignment for each peak for Area 1.

Peak Number	This Study (cm^−1^)	Past Study (cm^−1^)	Molecular Assignment
1	954	957–958	β-carotene [8,12,16]
2	980	984–985	Chlorophyll-a [12,13]
3	1002	1002–1006	β-carotene [7,12,17,18]
4	1017	1012–1016	Amino acid [19,20,21]

**Table 2:** Molecular assignment for each peak for Area 2.

Peak Number	This Study (cm^−1^)	Past Study (cm^−1^)	Molecular Assignment
5	1387	1389–1395	β-carotene [7,12,14,16]
6	1409	1416–1418	Cuticular Wax [15,22,23]
7	1454	1447–1456	Lycopene/β-carotene [14,16,24]

**Table 3:** Results of classification analysis.

Classifier	Predictor	Accuracy
Medium KNN	*P1i*, *P4i*, *P5p*, and *P7f*	90.9%
Weighted KNN		
Trilayered Neural Network		
Weighted KNN	*P1i*, *P1a*, *P3i*, *P3a*, *P4i*, *P4a*, *P5p*, *P5i*, *P7i*, *P7f*, *P7a*	81.8%
Discriminant Ensemble		

**Table 4:** Summary of classifiers, techniques and accuracy achieved for oil palm ripeness classification.

Classifier	Technique	Accuracy	Ref.
KNN	Sobel Edge Detection	65.0%	[30]
CNN	Image Processing	98.0%	[31]
ANN	Hyperspectral	95.0%	[6]
ANN	Image Processing	70.0%	[32]
LDA	Near Infrared	81.0%	[33]
SVM	Image Processing	98.9%	[2]
KNN	Raman Spectroscopy	90.9%	This study
